# The Opposite Effects of Kynurenic Acid and Different Kynurenic Acid Analogs on Tumor Necrosis Factor-α (TNF-α) Production and Tumor Necrosis Factor-Stimulated Gene-6 (TSG-6) Expression

**DOI:** 10.3389/fimmu.2019.01406

**Published:** 2019-06-21

**Authors:** Yvette Mándi, Valéria Endrész, Timea Mosolygó, Katalin Burián, Ildikó Lantos, Ferenc Fülöp, István Szatmári, Bálint Lőrinczi, Attila Balog, László Vécsei

**Affiliations:** ^1^Department of Medical Microbiology and Immunobiology, University of Szeged, Szeged, Hungary; ^2^Institute of Pharmaceutical Chemistry and Research Group for Stereochemistry, Hungarian Academy of Sciences, University of Szeged, Szeged, Hungary; ^3^Department of Rheumatology and Immunology, University of Szeged, Szeged, Hungary; ^4^Department of Neurology, University of Szeged, Szeged, Hungary

**Keywords:** kynurenic acid, TNF-α, TSG-6, U-937, *Staphylococcus*, *Chlamydia pneumoniae*

## Abstract

**Purpose:** The investigation of anti-inflammatory and immunosuppressive functions of Kynurenic acid (KYNA) is now in focus. There is also substantial evidence that TSG-6 has an anti-inflammatory activity. Therefore, in the present study, we compared the effects of newly synthetized KYNA analogs on the TNF-α production in U-937 monocytic cells in correlation with the effects on the TSG-6 expression.

**Methods:** TNF-α production was measured by ELISA, the TSG-6 expression was determined by RTqPCR method. As cytokine inducers *Staphylococcus aureus* and *Chlamydia pneumoniae* were used.

**Results:** KYNA and KYNA analogs attenuated TNF-α production and increased TSG-6 mRNA expression in U-937 cells stimulated by heat inactivated *Staphylococcus aureus*. In contrast, KYNA and some of the KYNA analogs increased the TNF-α production of *C. pneumoniae* infected U-937 cells; however, the newly synthetized analogs (SZR104, SZR 105, and SZR 109) exerted significant inhibitory effects on the TNF-α synthesis. The inhibitory and stimulatory effects correlated inversely with the TSG-6 expression.

**Conclusions:** TSG-6 expression following activation with bacterial components could participate in the suppression of inflammatory cytokines, such as TNF-α, We suppose that the elevation of the TSG-6 expression by KYNA and especially by new KYNA analogs might be one of the mechanisms that are responsible for their suppressive effect on TNF-α production as a feedback mechanism. KYNA and KYNA analogs have an important role in influencing TSG-6 expression, and there is a possible benefit of targeting TSG-6 expression by kynurenines in inflammatory conditions following infections.

## Introduction

There is an increasing interest in the role of kynurenines in the immune function. The kynurenine pathway is a regulator of both innate and adaptive immune responses, and the tryptophan metabolism kynurenine and production reflect a crucial interface between the immune and nervous systems (1, 2). Kynurenic acid (KYNA) is one of the products of the kynurenine pathway of tryptophan metabolism (3–5). KYNA as an antagonist of ionotropic glutamate receptors N-methyl-D-aspartate (NMDA) and the α7 nicotinic acetylcholine receptor (α7nAchR) exert neuroprotective effects (2, 4–10). KYNA acts both as a blocker of the glycine co-agonistic site of the NMDA receptor and as a non-competitive inhibitor of the α7 nicotinic acetylcholine receptor (11). The investigation of anti-inflammatory and immunosuppressive functions of KYNA is now in focus. It has been proved that these immunomodulatory properties are based on the signaling by G-protein-coupled receptor 35 (GP35) and aryl hydrocarbon receptor (AHR)-mediated pathways ys (2, 12–14).

Several studies have revealed that KYNA can attenuate inflammation induced by different stimuli (2, 15, 16). Previously, we demonstrated that KYNA and a KYNA analog reduced the TNF-α secretion from human mononuclear cells (17). In the present study, we compared the effects of newly synthetized KYNA analogs on the α TNF-α production in U-937 monocytic cell line. We focused on the potential correlation between the effects on the TSG-6 (TNFα- stimulated gene 6) expression and the influence, i.e., the suppression, of TNF-α production by different KYNA analogs.

Tumor necrosis factor -stimulated gene-6 (TSG-6) product is an 35-kDa hyaluronan(HA)-binding protein (18, 19) that is secreted by a wide range of cell types in response to inflammatory mediators. TSG-6 expression has been shown to be induced in fibroblasts, chondrocytes, monocytes, mesenchymal stem cells, vascular smooth muscle cells upon stimulation by proinflammatory signals (20). Moreover, TSG-6 is expressed by astrocytes in the brain (21). A substantial number of studies have shown that TSG-6 has anti-inflammatory activity (18, 20, 22–27).

TSG-6 has been reported to inhibit the association of TLR4 with MyD88, thereby suppressing NF-κB activation (26). TSG-6 has also prevented the expression of proinflammatory proteins (iNOS, IL-6, TNFα, IL-1β). TSG-6 functions by converting macrophages from a proinflammatory to an anti-inflammatory phenotype by suppression of TLR4/NF-κB signaling and STAT1 and STAT3 activation (26).The inhibition of the TLR2 pathway has also been reported (28).

Therefore, the aim of the present study is to evaluate a possible connection between the capacity of KYNA and KYNA analogs on the TSG-6 expression and the inhibition of TNF-α production first of all in U-937 monocytic cells. Our hypothesis was that activation of TSG-6 expression might be at least partially responsible for the TNF-α inhibitory effect of KYNA. TNF-α induction in U-937 cells was performed with heat killed *Staphylococcus aureus*, and the effects were compared with *Chlamydia pneumoniae (C. pneumoniae). Staphylococcus aureus* is a Gram-positive pyogenic coccus and a good inducer of TNF in mononuclear cells, and it mimics natural conditions (29, 30). *Chlamydia pneumoniae* is a Gram-negative bacterium, growing intracellularly, and it is responsible for different inflammatry conditions, especially in the lungs and in atherosclerosis. *Chlamydia pneumoniae* attach monocytes and multiply in them (31).The main question was, whether the production of TNF-α, and TSG-6 could be induced by these criteriae in U-937 cells. It was demonstrated in a previous study, that *C.pneumoniae* upregulated. numerous inflammatory genes in U-937 cells (32).

## Materials and Methods

### Reagents

KYNA (Kynurenic acid) was purchased from Sigma-Aldrich (Steinheim, Germany). Compounds SZR-72, SZR-73, and SZR-81 were synthesized by direct amidation of KYNA (33). In case of SZR-104, SZR-105, and SZR-109, the syntheses were achieved starting from the corresponding amides followed by C-3 aminoalkylation with morpholine or with diethylamine in the presence of formaldehyde (34, 35) (Table 1). KYNA and the analogs were dissolved in phosphate buffered saline (PBS) and added in increasing concentration in the μM range to the cell cultures.

**Table 1:** KYNA and KYNA analogs used in the experiments.

**Code**	**Structure**	**Chemical name**	**Empirical formula and Mw**
KYNA	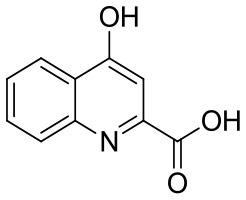	4-hydroxyquinolin-2-carboxylic acid	C_10_H_7_NO_3_ 189.17
SzR-72	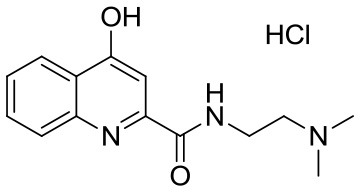	*N*-(2-(dimethylamino)ethyl)-4-hydroxyquinoline-2-carboxamide hydrochloride	C_14_H_18_ClN_3_O_2_ 295.76
SzR-73	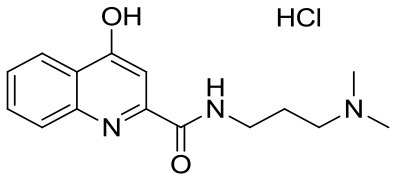	*N*-(3-(dimethylamino)propyl)-4-hydroxyquinoline-2-carboxamide hydrochloride	C_15_H_20_ClN_3_O_2_ 309.79
SzR-81	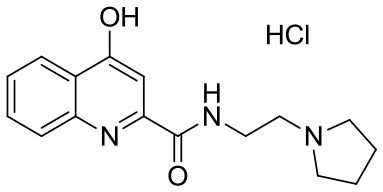	*N*-(2-(pyrrolidin-1-yl)ethyl)-4-hydroxyquinoline-2-carboxamide hydrochloride	C_16_H_20_ClN_3_O_2_ 321.80
SzR-104	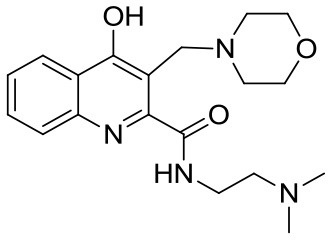	*N*-(2-(dimethylamino)ethyl)-3-(morpholinomethyl)-4-hydroxyquinoline-2-carboxamide	C_19_H_26_N_4_O_3_ 358.43
SzR-105	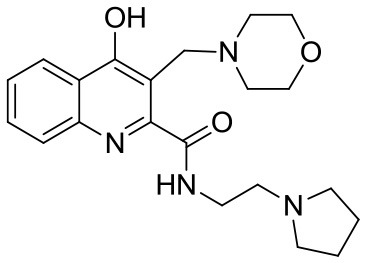	*N*-(2-(pyrrolidin-1-yl)ethyl)-3-(morpholinomethyl)-4-hydroxyquinoline-2-carboxamide	C_21_H_28_N_4_O_3_ 384.47
SzR-109	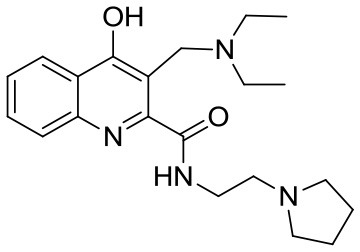	*N*-(2-(pyrrolidin-1-yl)ethyl)-3-((diethylamino)methyl)-4-hydroxyquinoline-2-carboxamide	C_21_H_30_N_4_O_2_ 370.49

### Cell Lines and Infection

U-937 cells were grown in RPMI 1640 medium supplemented with 10% heat-inactivated FBS (Biowest, Nuaille, France), 2 mmol/L L-glutamine, 1x nonessential amino acids, HEPES 4 mmol/L, 25 μg/mL gentamicin, and 0.5 μg/mL fungizone. HEp-2 cells were maintained in minimal essential medium (MEM) with Earle's salts completed with 10% FBS, 2 mmol/L L-glutamine, 1x nonessential amino acids, 25 μg/mL gentamicin, and 0.5 μg/mL fungizone. All reagents were purchased from SIGMA, St. Louis, MO, USA, unless otherwise indicated. The cell lines were purchased from ATCC. For TNF-α and TSG-6 induction, 5 × 10^5^ U-937 cells/mL were infected with 10^7^ heat inactivated *Staphylococcus aureus (S.aureus)*, or with 5 MOI (multiplicity of infection) *Chlamydia pneumoniae*. Cell supernatants were tested for TNF-α content by ELISA and cell lysates for TSG-6 mRNA by RT qPCR.

### Bacterial Strains

*Staphylococcus aureus* (*S. aureus*, SA1) 10^8^ /mL, were heat inactivated (29) and were used as a TNF-α inducer (30).

*Chlamydia pneumoniae (C. pneumoniae)* CWL029 strain from American Types Culture Collection (ATCC) was propagated in HEp-2 cells. Infective chlamydiae were quantitated by indirect immunofluorescent method applying anti-Chlamydia lipopolysaccharide (cLPS) monoclonal antibody (AbD Serotec, Oxford, United Kingdom) and FITC-labeled anti-mouse IgG (Sigma-Aldrich, St. Louis, MO). The concentration of infective elementary bodies (EB)-s was expressed as inclusion forming units/mL (IFU/mL).

### Stimulation of U 937 Cells by Bacteria Infection

(a) U-937 cells (5 × 10^5^ cells/mL) were stimulated with 10^7^ heat inactivated *S. aureus* (29) as a TNF inducer (30) and were incubated for 24 h in CO_2_ incubator at 37°C in complete RPMI. In parallel experiments, the cell cultures were pretreated for 30 min with KYNA and KYNA analoques at a concentration of 250–500 μM. In our prevous experiments (17), these concentrations proved to be optimal in reducing cytokine production. Cell supernatants were tested for TNF-α and TSG-6 content by ELISA and cell lysates for TSG-6 mRNA by RT qPCR.

(b) U-937 cells were seeded in 24-well plates (5 × 10^5^ cells/well), and the cells were then infected with *C. pneumoniae* at a multiplicity of infection (MOI) of 5 in complete RPMI with 0.5% glucose and centrifuged at 800 × g for 1 h RT. The growth medium was replaced in the wells with a medium containing KYNA analogs at a concentration of 250–500 μM. The culture plates were incubated for 24 h in CO_2_ incubator at 37°C. Cell supernatants were tested for TNF-α and TSG-6 content by ELISA and cell lysates for TSG-6 mRNA by RT qPCR.

### Chlamydial DNA Quantitation

For the quantitative assessment of chlamydial replication, a direct DNA quantitation method was used (36). The cells in the 96-well plates were infected with *C. pneumoniae* at a multiplicity of infection (MOI) of 5. After 24 and 48 h, the infected cells in 3 parallel wells were washed in the plates twice with 200 μL/well phosphate buffered saline (PBS). Then 100 μL Milli-Q water was added to the wells, and the plates were stored at −80°C. In order to free the DNA from the cells, two freeze-thaw cycles were applied. Thoroughly mixed lysates were used as templates directly for quantitative PCR (qPCR) using SsoFast™ EvaGreen® Supermix (BioRad). For the detection of *C. pneumoniae* DNA, the following primers were used: *ompA* F: 5′ TGCGACGCTATTAGCTTACGT 3′ and *ompA* R: 5′ TAGTTTGCAGCAGCGGATCCA 3′. A BLAST search was performed to check the specificity of the product target sequence of the primer sets. The primers were synthetized by Integrated DNA Technologies Inc. (Montreal, Quebec, Canada). During qPCR reaction, after the 10 min at 95°C polymerase activation step, 40 PCR cycles of 20 s at 95°C, and 1 min at 64°C were performed. The fluorescence intensity was measured at the end of the annealing–extension step. The specificity of amplification was confirmed by the melting curve analysis. For each PCR, the cycle threshold (Ct) corresponding to the cycle, where the amplification curve crossed the base line, was determined. The difference in Ct values detected in the samples incubated with KYNA and the analogs at a concentration of 250 and 500 μM compared to that of the untreated samples was calculated.

### TNF-α ELISA

The TNF-α concentrations in the supernatants were quantified by using the TNF-α ELISA kit (Legend Max BioLegend San Diego) according to the instructions of the manufacturer.

### TSG-6 ELISA

The TSG-6 concentrations in the supernatants were quantified by using the TSG-6 ELISA kit (SIGMA U.S.A. St. Louis) according to the instructions of the manufacturer.

### TSG-6 mRNA Quantification by Reverse Transcription Quantitative PCR (RT qPCR)

Total RNA was extracted from the samples by using TRI Reagent (Sigma-Aldrich, St. Louis, MO, USA) according to the manufacturer's protocol. The quality and the quantity of the extracted RNA were assessed by a NanoDrop Lite spectrophotometer (Thermo Scientific, Waltham, MA, USA). First-strand cDNA was synthesized by using 2 μg of total RNA with High-Capacity cDNA Reverse Transcription Kit (Applied Biosystems, Foster City, CA, USA) strictly adhering to the manufacturer's recommendations. The qPCR was conducted with cDNA, 1 μL of primers (10 μM) and SensiFast SYBR® No-ROX Mix (Bioline GmbH, Luckenwalde, Germany) in a total volume of 10 μL. The primers used in the assay were the following: TSG-6 sense 5′- ACT CAA GTA TGG TCA GCG TAT TC−3′, TSG-6 antisense 5′- GCC ATG GAC ATC ATC GTA ACT−3′; β-actin sense 5′- TTC TAC AAT GAG CTG CGT GTG GCT−3′, and β-actin antisense 5′- TAG CAC AGC CTG GAT AGC AAC GTA−3′. All primers were synthetized by Integrated DNA Technologies Inc. (Montreal, Quebec, Canada). The RT-qPCR was performed in a CFX96 Touch PCR detection system (Bio-Rad, Hercules, CA, USA). Thermal cycling was initiated with a denaturation step of 2 min at 95 °C followed by 40 cycles each of 10 s at 95°C and 1 min at 60°C. The fluorescence intensity was detected at the end of the annealing–extension steps. The specificity of amplification was confirmed by carrying out a melting curve analysis. The cycle threshold (C_t_) corresponding to the cycle, where the amplification curve crossed the base line, was determined. The Ct of target transcripts was compared with that of β-actin, the difference being referred to as ΔC_t_. The relative expression level was given as 2^−(ΔΔ*Ct*)^, where ΔΔC_t_ = ΔC_t_ for the experimental sample minus ΔC_t_ for the control sample. Increases in transcripts >2-fold compared to the control samples were considered to be significant. Uninfected cells were used as controls. All of the measurements were performed in duplicate from 3 biological repetitions.

### Human Blood Samples

EDTA-anticoagulated peripheral blood samples from 10 healthy volunteers were obtained.

Samples (1 mL each) were incubated in the presence of heat inactivated S, aureus for 18 hr. Parallel blood samples were pretreated for 30 min with KYNA and KYNA analogs at a concentration of 500 μM. Following the incubation period, the blood samples were centrifuged at 300 × g, and the supernatants were tested for TNF-α and TSG-6 content by ELISA.

For the experiments performed with the human blood we have the approval of the ethics commitee of the Medical Faculty of the University. of Szeged (ETT-TUKEB 905/PI/09). This study was conducted in full accordance with the tenets of Declaration of Helsinki (1964).

### Statistical Analysis

Data are expressed as means ± SD. Differences between group means were determined by the unpaired Student *t*-test. *p*-values < 0.05 were considered significant. Data of box and whiskers analysis were evaluated by Mann-Whitney test. The correlation between the TNF-α production and expression of TSG-6 was evaluated by correlation analysis. All statistical calculations were performed with the Graph-Pad Prism 5 statistical program (GraphPad Software Inc., San Diego, CA, USA).

## Results

### KYNA and KYNA Analogs Attenuate TNF-α Production in U-937 Human Monocytic Cells Stimulated by Heat Inactivated *Staphylococcus aureus*

**Figure 1:**
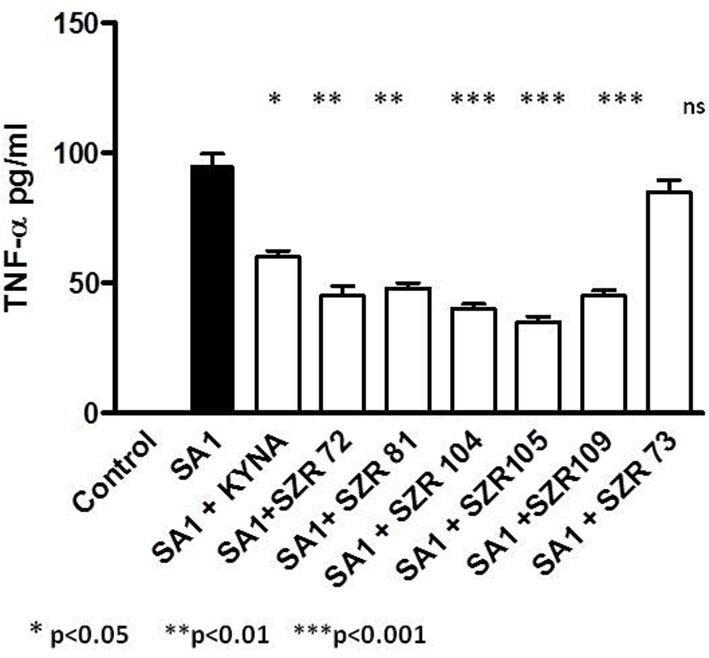
KYNA and KYNA analogs attenuate TNF-α levels in SA1 stimulated U-937 cells. U-937 cells (5 × 10^5^/ml) were stimulated with heat inactivated SA (107/ml) alone, (filled bar), or incubated together with KYNA or KYNA analogs at a concentration of 500 μM, which were added for 30 min prior to the addition of SA1 (open bars). The TNF-α levels in the supernatants were determined after 24 h by ELISA. Each concentration was tested in duplicate. Data are shown as means ± SD of three experiments. *p < 0.01; **p < 0.001 vs. the samples induced only with SA1 determined by the Student *t*-test.

The maximum TNF-α concentrations in the supernatants in SA1-induced cultures of U-937 cells without pretreatment of KYNA and derivates were 95. ± 8.5 pg/mL. At a concentration of 500 μM, all KYNA analogs suppressed the TNF-α level significantly, except SZR 73 (Figure 1). The new analogs SZR 104, 105, and 109 exerted the most potent inhibitory effects (*p* < 0.001) in equimolar (500 μM) concentration. Results obtained with 500 μM of the chemicals are demonstrated in Figure 1. In our previous experiments (17), 25 μM KYNA and SZR72 proved to be ineffective. At increasing concentrations (125, 250, and 500 μM), KYNA and SZR72 exhibited increasing inhibitory effects on TNF-α production. Similar results were obtained in the present experiments (data not shown), but only the result with the most effective concentration (500 μM) is demonstrated in this paper (Figure 1).

### KYNA and KYNA Analogs Increase TSG-6 mRNA Expression in U-937 Cells

**Figure 2:**
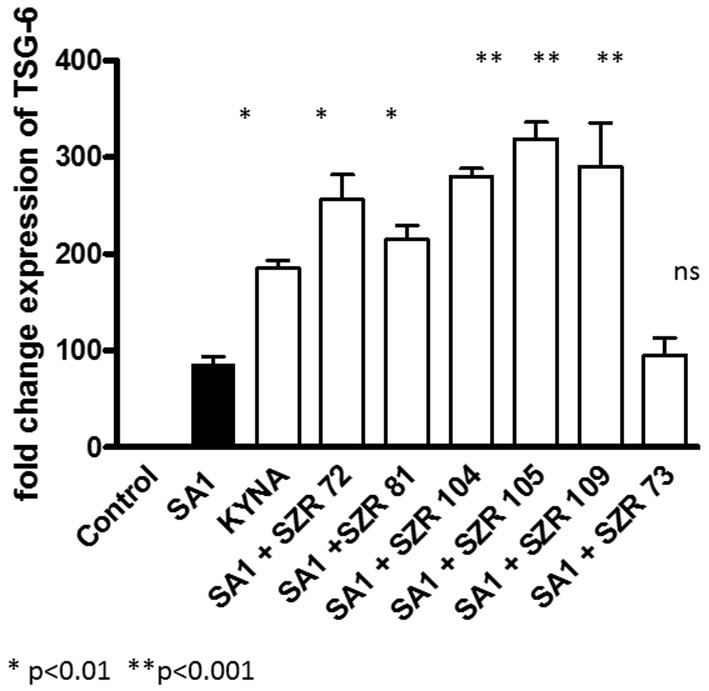
Effect of 500 μM of KYNA and KYNA analogs on TSG-6 mRNA levels in U-937 monocytic cells stimulated with SA1. TSG-6 expressions were normalized to reference gene of β-actin by using quantitative real-time PCR. Relative expression was calculated by using the 2^−(ΔΔ*Ct*)^ method and is given as a ratio between the target and the reference gene. Control: TSG-6 mRNA expression without stimulation, Filled bar: TSG-6 mRNA expression in SA1-stimulated cells without KYNA or KYNA analoques, open bars: TSG-6 mRNA expression in SA1-stimulated cells in the presence of 500 μM KYNA or KYNA analogs. Data are shown as means ± SD of the results of three independent experiments. *p < 0.01 vs. SA1 induced cells without chemicals, **p < 0.001 vs. SA1 induced cells without chemicals, determined by the Student *t*-test.

To gain further insight into the connection between the inhibition of TNF-α production and the induction of TSG-6 expression exerted by KYNA analoques, we determined the effects of KYNA analoques on TSG-6 mRNA expression. Both KYNA and KYNA analogs increased the TSG-6 relative expression at equimolar concentrations of 500 μM (Figure 2) significantly. SZR 73 was not effective in this respect, similarly as it was observed in the experiments with TNF-α production. Thus, we suspect that there is a connection between the attenuation of SA1-induced TNF protein synthesis and the TSG-6 gene transcription, which is elevated by KYNA and KYNA analoques.

### KYNA and the KYNA Analogs Differently Influence TNF-α Production Induced by *C. pneumoniae* in U-937 Human Monocytic Cells

**Figure 3:**
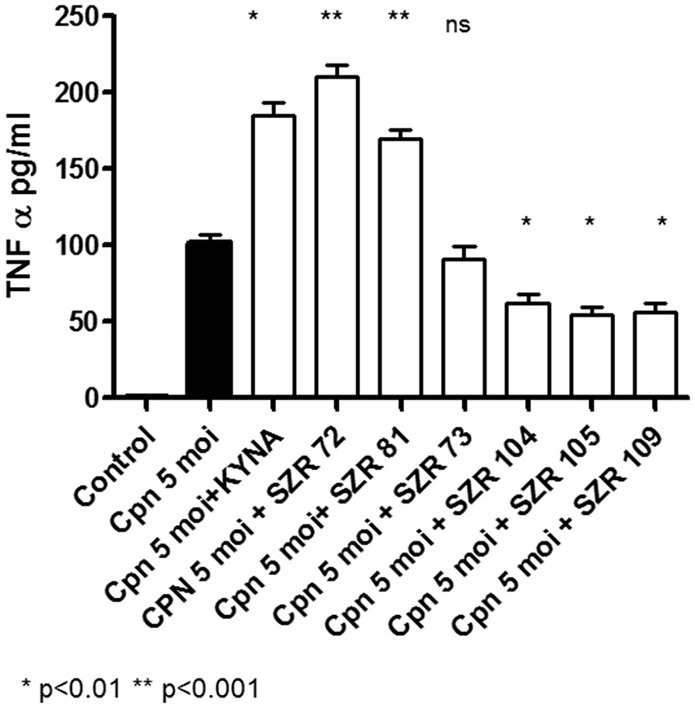
Effect of KYNA and KYNA analogs on TNF-α production in U-937 human monocytic cells stimulated by *Chlamydia pneumonia*. U-937 cells were pretreated for 30 min with KYNA or KYNA analogs and thereafter incubated for 24 h with 5 MOI of *Chlamydia pneumoniae*. U-937 cells (5 × 10^5^/mL) were infected with 5 MOI of *Chlamydia pneumoniae* alone (filled bar), or incubated together with KYNA or KYNA analogs at a concentration of 500 μM, which were added for 30 min prior to the addition of the bacteria (open bars). The TNF-α levels in the supernatants were determined after 24 h. Each concentration was tested in duplicate. Data are shown as means ± SD of three experiments. *p < 0.01; **p < 0.001 vs. the samples induced only with *C. pneumoniae* determined by the Student *t*-test.

We wanted to compare the effects of KYNA and KYNA derivates on TNF-α production when the inducer is a Gram-negative, intracellular bacterium, i.e*., Chlamydia pneumoniae (C. pneumoniae)*. Our results were unexpected; instead of having inhibitory effects, KYNA and some of the KYNA analoques increased the TNF-α production of *C. pneumoniae* infected U-937 cells. In contrast, the newly synthetized analogs (SZR104, SZR 105, and SZR 109) exerted a significant inhibitory effect on the cytokine synthesis (Figure 3).

### KYNA and KYNA Analogs Differently Influence TSG-6 mRNA Expression in U-937 Cells Infected With *Chlamydia pneumoniae*

**Figure 4:**
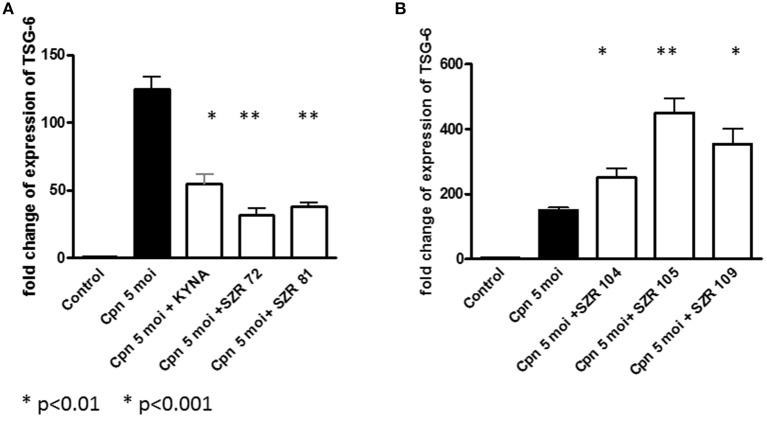
Effect of 500 μM of KYNA and KYNA analogs on TSG-6 mRNA levels in U-937 monocytic cells stimulated with *Chlamydia pneumoniae* at a MOI 5. TSG-6 expressions were normalized to the reference gene of β-actin by using quantitative real-time PCR. Relative expression was calculated by using the 2^−(ΔΔ*Ct*)^ method and is given as a ratio between the target and the reference gene. Control: TSG-6 mRNA expression without stimulation, Filled bar: TSG-6 mRNA expression in *C. pneumoniae*-stimulated cells without KYNA or KYNA analoques, open bars: TSG-6 mRNA expression in *C. pneumoniae*-stimulated cells in the presence of 500 μM KYNA or KYNA analoques. Data are shown as means ± SD of the results of three independent experiments. *p < 0.01 vs. *C. pneumoniae*- induced cells without chemicals, **p < 0.001 vs. *C. pneumoniae*-induced cells without chemicals, determined by the Student *t*-test. **(A)** Decreasing, **(B)** Increasing effects.

**Figure 5:**
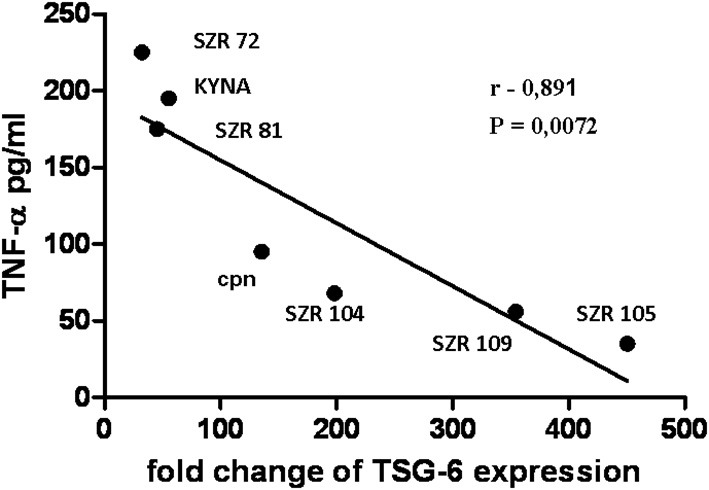
Correlation between the TSG-6 expression and TNF-α production by U-937 cells infected with *C. pneumoniae* at a MOI 5 in the presence of KYNA or the analogs. U-937 cells were pretreated for 30 min with KYNA or KYNA analogs at a concentration of 500 μM, and thereafter incubated for 24 h with 5 MOI of *Chlamydia pneumoniae*. The TNF-α levels in the supernatants were determined with ELISA assay, and the TSG-6 expression by RT qPCR reactions. The significance of correlation was calculated by correlation analysis with the Graph-Pad Prism 5 statistical program. Symbols and numbers represent the data obtained with KYNA or KYNA analogs. Cpn: incubation only with *Chlamydia pneumoniae* without compounds. The correlation coefficient, r value is-0.891, the *p*-value = 0.0072, the 95% confidence interval is −0.9838 to −0,4174.

*C. pneumoniae* induced a considerable TSG-6 expression in U-937 cells. KYNA, SZR72, and SZR81 inhibited the rate of expression (Figure 4A). Interestingly, the same chemicals enhanced the TNF-α production of *C. pneumoniae-*induced U-937 cells (Figure 3). On the other hand, further KYNA analoques (SZR 104, SZR 105, and SZR 109) with different chemical structure (see Table 1) stimulated TSG-6 expression (Figure 4B). It is also noteworthy that only these analoques inhibited significantly the TNF-α production of *C. pneumoniae-*induced U-937 cells (Figure 3). Considering the variable effects of KYNA analogs on the TSG-6 expression and also on the TNF-α production, we checked the correlation between the two effects. As it was expected, a significant inverse correlation was found between the effects on the TNF-α secretion and the TSG-6 expression exerted by different KYNA analogs (Figure 5). KYNA, SZR72, and SZR81 induced higher TNF-α secretion by U-937 cells after *C. pneumoniae* infection, but they decreased the TSG-6 expression compared to the cells that were infected only with *C. peumomiae*, without any of the compounds (i.e., Cpn in Figure 5). In contrast, in the case of the highest rate of TSG-6 expression (SZR 105), a maximal rate of inhibition of TNF-α production was observed. Therefore, we suppose that the different effects of KYNA analoques on the TSG-6 expression in *C. pneumoniae* infected cells might explain the difference in their effects on the secretion of TNF-α.

Altogether, from these data, it seems that inhibition of TNF-α is not only in correlation with the antiinflammatory effect of TSG-6, but in this situations, KYNA analogs are able to increase or even decrease the expression of TSG-6.

### Effects of KYNA Analoques on the Quantity of *C. pneumoniae*

**Figure 6:**
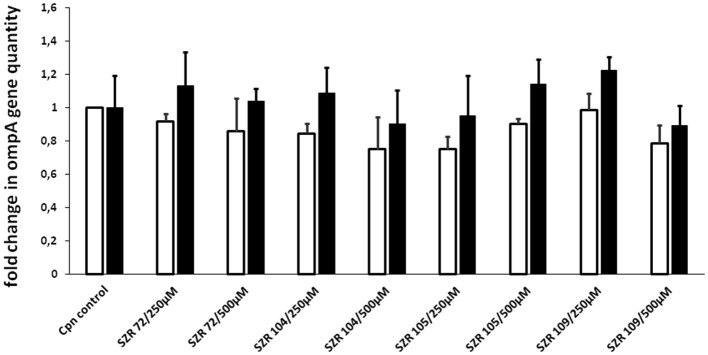
Analysis of the effect of kynurenic acid derivates on *C. pneumoniae* growth in U-937 cells based on quantitation of chlamydial DNA by qPCR. The cells were infected in 96-well plates at a MOI of 5 in a medium containing kinurenic acid derivates. Direct detection of *C. pneumoniae ompA* gene in the lysate of infected cells was performed at 24 (open bars) and 48 h (filled bars) postinfection. Fold change in the quantity of chlamydial DNA in kinurenic acid derivate treated cultures compared to the quantities detected in non-treated cultures was calculated. The mean of fold change in 3 parallel cultures and SD are shown. The differences are not significant.

To ascertain that the effects of KYNA analoques on the TNF-α or TSG-6 induction is not simply due to their effects on the replication of *C. pneumoniae*, we performed experiments for quantitative assessment of chlamydial replication by a direct quantitative PCR method (36). *C. pneumoniae* ompA gene was detected in the lysate of U-937 cells infected with *C. pneumoniae* at a MOI of 5 in the presence or absence of KYNA analoques at a concentration of 250 or 500 μM, respectively. Direct detection of *C. pneumoniae* DNA in the lysate of infected cells was done at 24 and 48 h postinfection. There was no significant inhibition or even elevation in the quantity of chlamydial DNA in the presence of different KYNA analoques after the 24 h (open bars) or 48 h (filled bars) incubation period. The results of the samples tested at 24 and 48 h of incubation are presented in Figure 6. Therefore, we assume that KYNA analoques do not influence the replication or the quantity of *C. pneumoniae*.

### Effects of KYNA Analogs on TGS-6 Protein Production in U-937 Human Monocytic Cells Stimulated With Heat Inactivated *S. aureus* or by *Chlamydia pneumoniae*

To ascertain whether the effects of KYNA and analogs on the TSG-6 expression influence parallelly the protein level, the TSG-6 concentrations in the supernatants of U-937 cells were determined.

**Figure 7:**
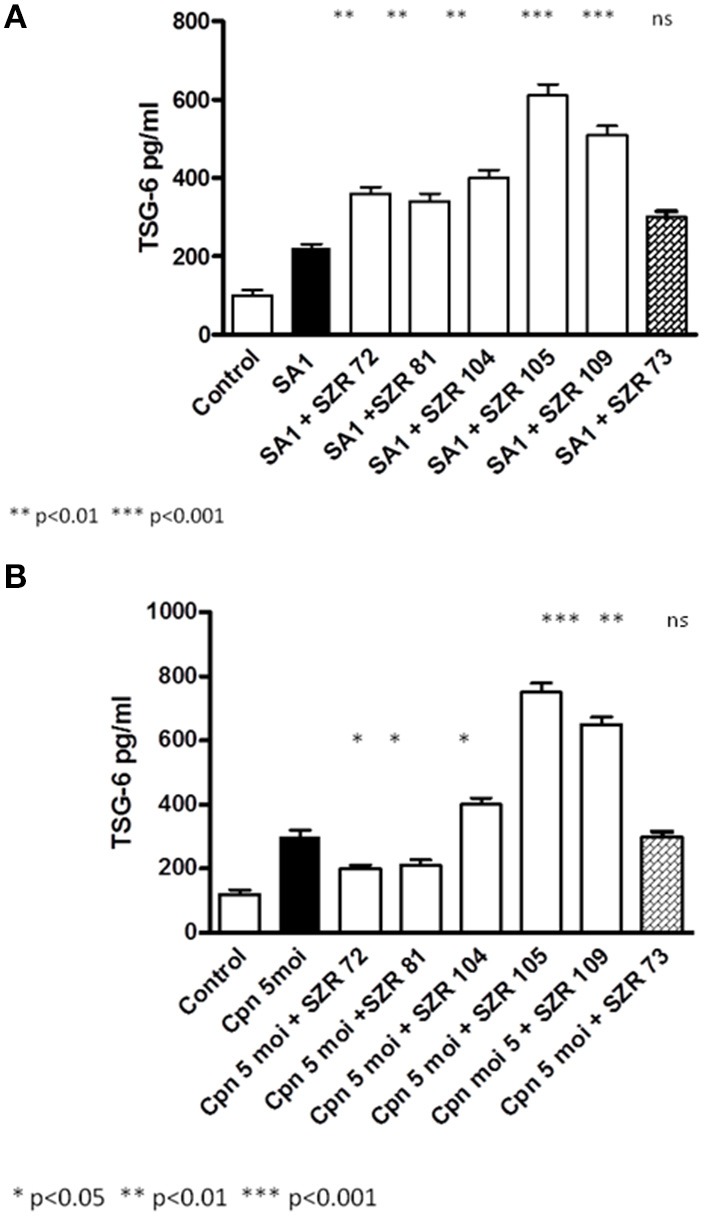
Effect of KYNA and KYNA analogs on TGS-6 protein production in U-937 human monocytic cells stimulated with heat inactivated *S. aureus* (SA1) **(A)** or by *Chlamydia pneumoniae* **(B)**. **(A)** U-937 cells (5 × 10^5^/ml) were stimulated with heat inactivated SA1 (10^7^/ml) alone, (filled bar), or incubated together with KYNA or KYNA analogs at a concentration of 500 μM, which were added for 30 min prior to the addition of SA1 (open bars). The TSG-6 levels in the supernatants were determined after 24 h by ELISA. Each concentration was tested in duplicate. Data are shown as means ± SD of three experiments. *p < 0.01; **p < 0.001 vs. the samples induced only with SA1 determined by the Student *t*-test. **(B)** U-937 cells were pretreated for 30 min with KYNA or KYNA analogs and thereafter incubated for 24 h with 5 MOI of *Chlamydia pneumoniae*. U-937 cells (5 × 10^5^/mL) were infected with 5 MOI of *Chlamydia pneumoniae* alone (filled bar), or incubated together with KYNA or KYNA analogs at a concentration of 500 μM, which were added for 30 min prior to the addition of the bacteria (open bars). The TSG-6 levels in the supernatants were determined after 24 h. Each concentration was tested in duplicate. Data are shown as means ±SD of three experiments. *p < 0.01; **p < 0.001 vs. the samples induced only with *C. pneumoniae* determined by the Student *t*-test.

At a concentration of 500 μM, KYNA and KYNA analogs increased the TGF-6 level significantly, except SZR 73 in SA1 induced cells (Figure 7A). The new analogs SZR 104, 105, and 109 exerted the most potent stimulatory effects (*p* < 0.001) in equimolar (500 μM) concentration. *C. pneumoniae* induced also TSG-6 production in U-937 cells, but KYNA, SZR72, and SZR81 decreased the level of TSG-6 protein expression (Figure 7B). On the other hand, further KYNA analoques (SZR 104, SZR 105, and SZR 109) increased the TSG-6 concentration in the supernatants (7b). These experiments obtained with 500 μM of KYNA and KYNA analogs support the results obtained with RT PCR data demonstrating the effects of the chemicals on the TSG-6 RNA expression.

### KYNA Analogs SZR 72 and SZR 105 Attenuate TNF-α Production and Increase TSG-6 Secretion in Human Whole Blood Cells Stimulated by Heat Inactivated *Staphylococcus aureus*

Some of the results obtained by *in vitro* experiments with U-937 monocytic cells were repeated by “*ex vivo*” experimets investigating the effects of two KYNA analogs in human peripheral blood.

**Figure 8:**
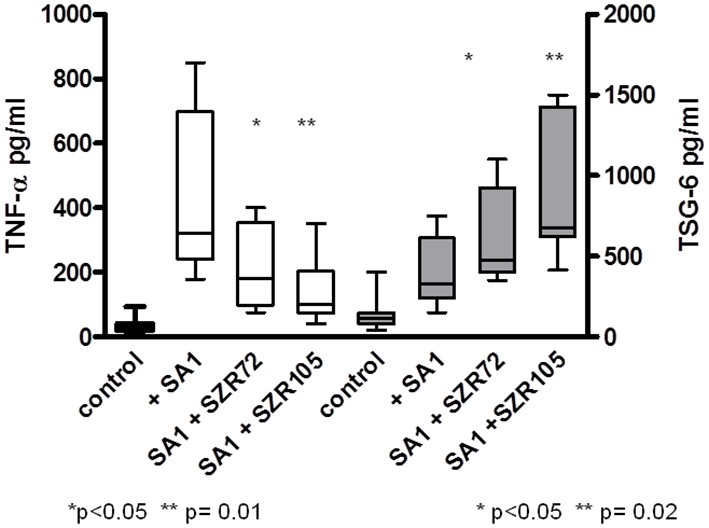
Effect of KYNA analogs SZR 72 and SZR 105 on the TNF-α production and TSG-6 secretion in human whole blood cells stimulated by heat inactivated *Staphylococcus aureus*. EDTA-anticoagulated blood samples 1–1 mL each of 10 donors were incubated with SZR72 or SZR105 at a concentration of 500 μM for 30 min prior to the addition of heat inactivated *Staphylococcus aureus* (10^7^/ml) The concentrations of TNF-α and that of TSG-6 in the plasma were determined after 18 h incubation period by TNF-α and TSG-6 ELISA plotted on the left and right Y axis, respectively. The data are depicted as box and whiskers plots, where the lines inside the boxes denote medians, and the boxes mark the interval between 25 and 75 percentile, and the whiskers the maximum and minimum. Significance were determined by the Mann-Whitney test.

There was big individual differences in the TNF-α concentrations and in TSG-6 concentrations in the supernatants in SA1-induced blood cultures (Figure 8), between 179 pg/ml and 850 pg/ml, and between 150 and 750 pg/ml, respectively. At a concentration of 500 μM, both SZR 72 and SZR 105 suppressed the TNF-α level significantly in the S. aureus induced blood cultures. Again, the new analog SZR 105 exerted more potent inhibitory effect (*p* = 0.001) in equimolar (500 μM) concentration. Similarly to the effects on U-937 cells, the KYNA analogs SZR72 and SZR 105 significantly increased the TSG-6 concentrations in SA1 induced blood samples (Figure 8).

## Discussion

In our experiments, KYNA and different KYNA derivates inhibited the TNF-α production of U-937 cells stimulated with heat inactivated *Staphylococcus aureus*. The rate of the inhibition was variable according the structure of the analoques (Figure 1). The effect of the analogs were compared in equimolar concentration on the TNF-α production when the inducer was *Chlamydia pneumoniae*, a Gram negative, intracellular bacterium. In these experiments, however, not all KYNA derivates inhibited TNF-α production by U-937 monocytic cells; moreover, KYNA itself, and SZR72 and SZR81 increased it (Figure 3). We hypothesized that the difference in the influence on the TNF-α production might be connected with the difference in the TSG-6 expression (Figure 4).

The production of TNF-α in *C. pneumoniae* infected cells was inhibited only by the KYNA derivates (SZR 104, SZR105, SZR109) that upregulated the expression of TSG-6 (Figures 4, 5).

It is noteworthy, that TSG-6 itself does not only exert an antiinflammatory effect (20, 26, 27), but its expression might be under the influence of KYNA (37). It has been published that kynurenic acid controls TSG-6-mediated immunosuppression of the human mesenchymal stem cells (MSCs). In elegant experiments, it has been demonstrated that KYNA specifically regulates TSG-6 production by activating aryl hydrocarbon receptor (AHR). KYNA activates AHR, which directly binds to the TSG-6 promoter to enhance TSG-6 expression. Moreover, KYNA-pretreated MSCs can further boost TSG-6 production, and thus enhance the therapeutic capacity of human MSCs against lipopolysaccharide (LPS)-induced acute lung injury (37).

We found that in most experiments, TSG-6 expression was up-regulated in U-937 monocytic cells stimulated with bacterial components, and KYNA and KYNA analogs were able to influence the rate of expression of TSG-6. The elevation of the TSG-6 expression might be one of the mechanisms that are responsible for the suppression of TNF-α production as a feedback effect. This effect was clearly demonstrated in our experiments using heat inactivated *S. aureus* as a cytokine inducer. In the case of *C. pneumoniae* infection, however, KYNA and KYNA analoques did not exert this effect uniformly. Some of them increased TSG-6 expression with a concomitant inhibition of the production of TNF-α, but several compounds (KYNA, SZR72, and SZR 81) rather decreased the expression of TSG-6, and it is very likely that this could lead to an elevated TNF-α production compared to the TNF-α production of U-937 cells infected with *C. pneumoniae* without any KYNA analoque. We hypothesized that the explanation of the difference in the results might be due to the different chemical structure of the analoques (see Table 1). The examined substrates (SZR-72, SZR-73 SZR-81, SZR-104, SZR-105, and SZR-109) can be classified into two classes of compounds: the first are amide derivatives (SZR-72, SZR-73, SZR-81) containing cationic center at the amide side chain. The second class of compounds (SZR-104, SZR-105, and SZR-109) are the C-3 aminoalkylated amides, therefore they can be interpreted as derivatives with dual cationic centers.

They could differently influence the binding of *C. pneumoniae* to the Toll-like receptor 2 (TLR2), and especially, differently activate AHR in the presence of *C. pneumoniae*. It has to be highlighted that the newly synthetized analogs, SZR 105 and SZR 109, were the most potent inducers of TSG-6 expression, and the highest inhibitors of TNF-α production in both types of bacterial inducers. The study of the exact effect of *Chlamydia pneumoniae* on the interaction between AHR and some KYNA analogs needs to be further investigated and proved.

Whatever the explanation is, our results indicate that there is a close connection between TNF production and TSG-6 expression, and there is an inverse correlation between the TSG-6 expression and TNF-α production in the presence of KYNA and KYNA analogs.

This negative correlation was further demonstrated at the protein level of TSG-6 measured in the supernatants of U-937 cells. and also in unseparated human peripheral blood samples

The stimulation of TSG-6 expression by KYNA and KYNA derivates might be one of the mechanisms that have an important role in their suppressive effect on TNF-α production. TSG-6 expression following activation with bacterial components could participate in the suppression of inflammatory cytokines, such as TNF-α, and it is noteworthy that KYNA and especially KYNA analogs are able to enhance this effect. Further studies are required to elucidate the different effects of KYNA derivates in the case of different bacterial inducers and the possible benefits of targeting TSG-6 expression by kynurenines in inflammatory conditions following infections.

## Data Availability

All datasets generated for this study are included in the manuscript and/or the supplementary files.

## Ethics Statement

For the experiments performed with the human blood we have the approval of the ethics commitee of the Medical Faculty of the University of Szeged (ETT-TUKEB 905/PI/09). This study was conducted in full accordance with the tenets of Declaration of Helsinki (1964).

## Author Contributions

YM designed research and wrote the manuscript. VE, KB, and IL conducted experiments with *Chlamydia*. TM performed experiments with RT-PCR. FF, IS, and BL contributed new reagents. AB provided the blood samples, YM and VE analyzed data. LV organized research for neurological project. All authors read and approved the manuscript.

### Conflict of Interest Statement

The authors declare that the research was conducted in the absence of any commercial or financial relationships that could be construed as a potential conflict of interest.
